# Severe acute kidney disease is associated with worse kidney outcome among acute kidney injury patients

**DOI:** 10.1038/s41598-022-09599-7

**Published:** 2022-04-20

**Authors:** Yu-Wei Chen, Mei-Yi Wu, Cheng-Hsien Mao, Yu-Ting Yeh, Tzu-Ting Chen, Chia-Te Liao, Cai-Mei Zheng, Yung-Ho Hsu, Yih-Giun Cherng, Mai-Szu Wu

**Affiliations:** 1 Division of Nephrology, Department of Internal Medicine, Shuang Ho Hospital, Taipei Medical University, No. 291, Zhongzheng Road, Zhonghe District, New Taipei City, 235 Taiwan; 2 Graduate Institute of Clinical Medicine, College of Medicine, Taipei Medical University, Taipei, Taiwan; 3 TMU Research Center of Urology and Kidney, Taipei Medical University, Taipei, Taiwan; 4 Institute of Epidemiology and Preventive Medicine, College of Public Health, National Taiwan University, Taipei, Taiwan; 5 Division of Nephrology, Department of Internal Medicine, School of Medicine, College of Medicine, Taipei Medical University, Taipei, Taiwan; 6 Information Technology Office, Shuang Ho Hospital, Taipei Medical University, Taipei, Taiwan; 7 Center for Neuropsychiatric Research, National Health Research Institutes, Miaoli County, Taiwan; 8 Department of Anesthesiology, Shuang Ho Hospital, Taipei Medical University, New Taipei City, Taiwan; 9 Department of Anesthesiology, School of Medicine, College of Medicine, Taipei Medical University, Taipei, Taiwan

**Keywords:** Health care, Medical research, Risk factors

## Abstract

Acute kidney disease (AKD) comprises acute kidney injury (AKI). However, whether the AKD staging system has prognostic values among AKI patients with different baseline estimated glomerular filtration (eGFR) remains a controversial issue. Algorithm-based approach was applied to identify AKI occurrence and to define different AKD stages. Risk ratio for major adverse kidney events (MAKE), including (1) eGFR decline > 35% from baseline, (2) initiation of dialysis, (3) in-hospital mortality of different AKD subgroups were identified by multivariable logistic regression. Among the 4741 AKI patients identified from January 2015 to December 2018, AKD stages 1–3 after AKI was common (53% in the lower baseline eGFR group and 51% in the higher baseline eGFR group). In the logistic regression model adjusted for demographics and comorbidities at 1-year follow-up, AKD stages 1/2/3 (AKD stage 0 as reference group) were associated with higher risks of MAKE (AKD stage: odds ratio, 95% confidence interval [95% CI], AKD 1: 1.85, 1.56–2.19; AKD 2: 3.43, 2.85–4.12; AKD 3: 10.41, 8.68–12.49). Regardless of baseline eGFR, staging criteria for AKD identified AKI patients who were at higher risk of kidney function decline, dialysis and mortality. Post-AKI AKD patients with severer stage need intensified care and timely intervention.

## Introduction

Acute kidney injury (AKI) occurs in approximately 10–15% of hospitalized patients; however, its incidence in the intensive care unit can be more than 50% of patients^1^. Previous meta-analyses and systematic reviews have demonstrated that AKI might significantly increase the risk and progression of chronic kidney disease (CKD) as well as the risk of end-stage kidney disease (ESKD) and even mortality^2–6^. However, a recent prospective study determined that AKI stages were not independently associated with adverse clinical outcomes after adjustment for multiple CKD risk factors^7^.

Acute kidney disease (AKD), as an intermediary stage between AKI (abrupt deterioration of renal function within a period of 7 days or less) and CKD (persistent renal function impairment or structural abnormalities for more than 90 days), indicates changing renal function that could be strongly associated with long-term renal outcomes and could serve as a valuable indicator of the appropriate time window for therapeutic interventions^2,8,9^. A large population-based observational study discovered a statistically significant association between AKD and major adverse kidney events (MAKEs) related to CKD progression, ESKD, and mortality^10^. With or without AKI, AKD was also found to be associated with adverse clinical outcome among hospitalized patients^11^.

Nevertheless, the disparate definitions of AKD might indicate variance in the acuity and severity of kidney disease among different subgroups of AKD patients^12^. Persistent kidney dysfunction for more than 7 days after AKI (post-AKI AKD) is associated with adverse renal and cardiovascular outcomes^11,13–17^. The 16th Acute Disease Quality Initiative (ADQI) recommends definitions and staging criteria for post-AKI AKD and renal recovery, but the clinical trajectories of renal function after AKI and the prognostic value of AKD staging remain largely unknown^2,18^. Therefore, to explore the evolutional pattern of renal function following AKI, we obtained all of the available serum creatinine (SCr) data from a single tertiary hospital to define the incidence and clinical outcomes of different stages of post-AKI AKD.

## Methods

### Data source

We retrieved laboratory and administrative data from the health information system database of a single tertiary hospital to conduct a retrospective cohort study. The Joint Institutional Review Board of Taipei Medical University (TMU-JIRB-N201906062) approved the present study and waived the need for informed consent because all data had been de-identified. The present study was conducted in accordance with the Strengthening the Reporting of Observational Studies in Epidemiology (STROBE) guidelines^19^.

### Cohort information

All available SCr data of patients older than 20 years from January 1, 2014, to December 31, 2019, were statistically analyzed. Patient SCr data from January 1, 2015, to December 31, 2018, were first grouped into *per capita* data as an observational cohort. SCr data from January 1, 2014, to December 31, 2014, were all used as the source of the baseline SCr data pool (see “Definition and staging of AKI, AKD, and baseline eGFR classification”). SCr data and other laboratory data from January 1, 2019, to December 31, 2019, were included to ensure that the observational period of each patient with AKI identified by the algorithm would be equal to or more than 1 year. In addition, to explore the post-AKI prognostic value and clinical effect, we defined the date of AKI occurrence as the index date. Patients who did not develop AKI or who received their first dialysis before the index date were excluded. Moreover, patients younger than 20 years and those with missing information regarding date of birth or sex were excluded. Patients without data after AKI, without data within 7–90 days after AKI, and those without data beyond 90 days after AKI were also excluded to delineate the evolutional changes of renal function.

Underlying comorbidities, such as hypertensive heart disease, diabetes mellitus, anemia, cerebrovascular disease, malignancy, chronic obstructive pulmonary disease, and digestive tract disease, were identified using ICD-9-CM codes if they had been used one or more times for inpatient diagnoses or one or more times for outpatient diagnoses within 1 year before the occurrence of AKI (determined according to the creatinine index [C1]). Furthermore, to evaluate the possible prognostic effects of electrolytes, we averaged all available serum sodium and potassium data within 3 months before the occurrence of AKI and used the mean values as biochemical factors in our model.

### Definition and staging of AKI, AKD, and baseline eGFR classification

We defined AKI based on the 2014 NHS England “algorithm for detecting acute kidney injury (AKI) based on serum creatinine changes with time” (Fig. S1) to allow international comparisons with other studies on AKI electronic alert systems. The algorithm complied with the Kidney Disease: Improving Global Outcomes (KDIGO) guidelines regarding the definition of AKI based on SCr values. In this algorithm, C1 values served as the first input data. The lowest SCr values within 0 to 7 days before C1 were defined as the baseline creatinine data (RV1). If RV1 were not available, then the median value of all the available SCr data within 8–365 days before the C1 values were obtained were used as the baseline SCr (RV2). The index SCr-to-baseline SCr ratio (C1/RV1 or C1/RV2) was calculated to define AKI.

**Figure 1:**
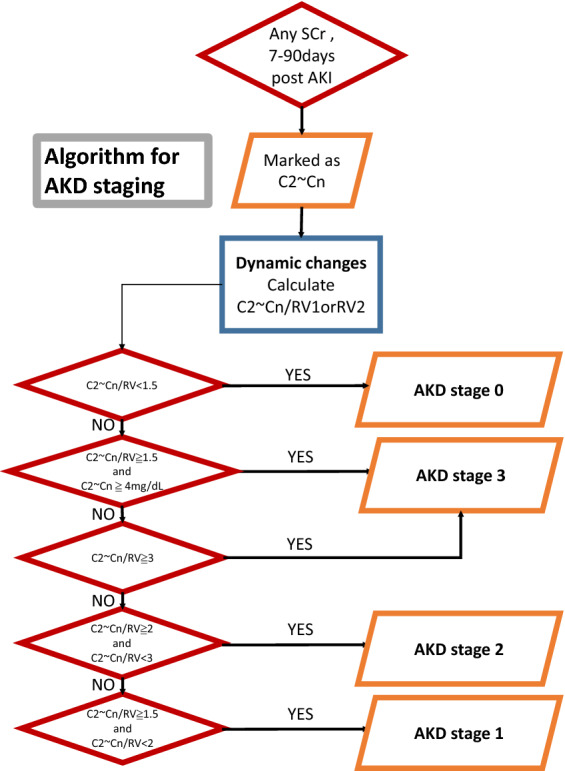
Algorithm for the staging of acute kidney disease. Algorithm for the staging of acute kidney disease (AKD), based on the 16th Acute Disease Quality Initiative (ADQI) consensus, which is congruent with AKI staging. *SCr* serum creatinine, *RV* reference value. Patients with AKD stage 0 by this algorithm were considered as reference group in the following statistical analysis. Conversion factors for serum creatinine in mg/dL to μmol/L, × 88.4.

Once AKI was defined, the creatinine data with the maximum values between the 7th and 90th day after AKI were retrieved to calculate the AKD stages by using another algorithm (Fig. 1). The AKD staging algorithm was based on the 16th ADQI consensus^2^, which is congruent with AKI staging. The patients were then categorized according to the worst AKD stages into AKD stage 0 (reference group) and AKD stage 1–3 (comparison groups).

Baseline eGFR classification were defined based on the estimated glomerular filtration rate (eGFR) derived from the reference SCr (RV1 or RV2), calculated using the Chronic Kidney Disease Epidemiology Collaboration equation^20^. To determine the clinical significance of AKI and AKD among the patients with different baseline eGFR, we categorized our cohort into two groups: “higher baseline eGFR group” and “lower baseline eGFR group” who had baseline eGFR ≥ 60 mL/min/1.73 m^2^ and baseline eGFR < 60 mL/min/1.73 m^2^, respectively.

### Follow-up and study outcomes

All patients with creatinine values were followed up for up to 365 days after AKI in this retrospective cohort study. We examined MAKEs as a composite end point, which included deterioration of renal function, dialysis, and in-hospital mortality. The outcomes were defined as follows: (1) “Deterioration of renal function” was defined as more than 35% decline in eGFR, comparing the eGFR at different time points with the baseline value. (2) “In-hospital mortality” was defined using the corresponding codes in the health information system. (3) “Dialysis” was defined as the first-time use of kidney replacement therapy (KRT) according to the relevant procedural code. Moreover, to evaluate whether AKD increases the burden on health care systems, we defined patients as receiving “prolonged dialysis” if they underwent dialysis more than 24 times within 3 months.

### Statistical analysis

We compared baseline characteristics according to the baseline eGFR classification using a *t* test for continuous variables and Chi-squared test for categorical variables. We used a logistic regression model to examine the prognostic effects of clinical indicators, including age, sex, comorbidities, baseline eGFR classification, AKI stages, and AKD stages. Basic demographic characteristics and comorbidities were adjusted. All analyses were performed using SAS statistical software (SAS System for Windows, version 9.4, SAS Institute Inc., Cary, NC, USA).

### Ethics approval

The study was approved by the Joint Institutional Review Board of Taipei Medical University (TMU-JIRB-N201906062) and the need for informed consent was waived because all data had been de-identified.

## Results

### Demographics and AKD incidence among patients with AKI

**Figure 2:**
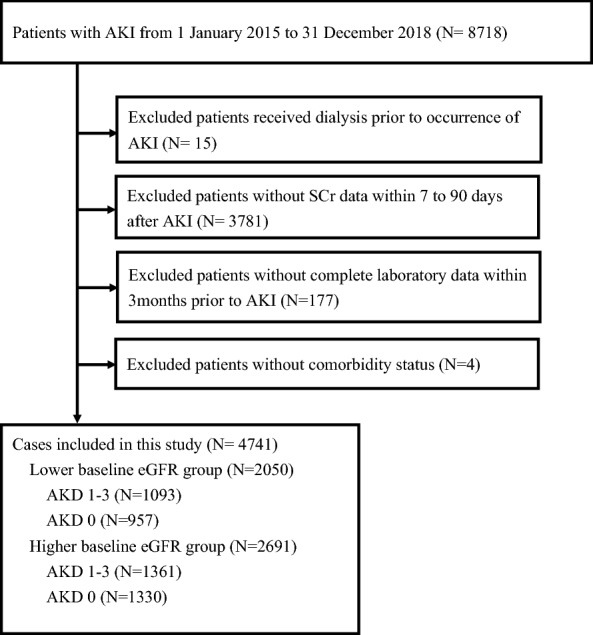
Flowchart demonstrating the inclusion of eligible patients. Flowchart demonstrates the selection criteria and process of eligible acute kidney injury patients for investigating the risk of major adverse kidney events. *AKI* acute kidney injury, *SCr* serum creatinine, *eGFR* estimated glomerular filtration rate, *AKD* acute kidney disease.

We identified 8718 patients with AKI between January 1, 2015 and December 31, 2018. However, 15 patients who underwent dialysis before AKI occurrence, 3781 patients without SCr data between 7 and 90 days after AKI, and 181 patients with incomplete laboratory data or ambiguous comorbidity status were excluded (Fig. 2). The remaining 4741 patients with AKI were all considered AKD patients, according to the KDIGO consensus^15^. These post-AKI AKD patients were then further categorized according to their baseline eGFR classification. Of these patients, 2050 had baseline eGFR < 60 mL/min/1.73 m^2^ before AKI (lower baseline eGFR group) and 2691 had baseline eGFR ≥ 60 mL/min/1.73 m^2^ before AKI (higher baseline eGFR group). The patient demographics for each group are listed in Table 1. The lower baseline eGFR group included 1093 patients who had AKD stage 1–3 after AKI (53.3%) and 957 patients who had AKD stage 0 after AKI. Nevertheless, no significant differences were noted between the AKD stage 1–3 and AKD stage 0 groups regarding age, sex, or percentages of patients with comorbidities. The higher baseline eGFR group included 1361 patients who had AKD stage 1–3 after AKI (50.5%) and 1330 patients who had AKD stage 0 after AKI. Compared with the AKD stage 0 group, the AKD stage 1–3 group had a significantly higher proportion of patients with anemia (7.9% versus 5.3%, *P* = 0.0070) and malignancy (41.0% versus 31.2%, *P* < 0.0001), but a lower proportion of patients with hypertension or cerebrovascular disease. The proportions of patients with different AKD stages within the lower baseline eGFR and the higher baseline eGFR groups are presented in Table 1, Table S1 and Table S2.

**Table 1:** Characteristics of AKI patients with different baseline eGFR classification and AKD status.

	Lower baseline eGFR			Higher baseline eGFR		
	AKI			AKI		
	AKD stage 0	AKD stage 1–3	P value	AKD stage 0	AKD stage 1–3	P value
N	957	1093		1330	1361	
Age, y, mean ± SD	74.9 ± 13.1	74.3 ± 14.0	0.275	65.7 ± 15.3	64.9 ± 15.3	0.198
**Age group, y, N (%)**			0.365			0.521
20–29	6 (0.6)	5 (0.5)		20 (1.5)	21 (1.5)	
30–39	10 (1.0)	19 (1.7)		50 (3.8)	49 (3.6)	
40–49	26 (2.7)	42 (3.8)		136 (10.2)	143 (10.5)	
50–59	71 (7.4)	92 (8.4)		241 (18.1)	287 (21.1)	
60–69	179 (18.7)	196 (17.9)		314 (23.6)	324 (23.8)	
70–79	267 (27.9)	273 (25.0)		288 (21.7)	264 (19.4)	
≥ 80	398 (41.6)	466 (42.6)		281 (21.1)	273 (20.1)	
Male sex, N (%)	502 (52.5)	587 (53.7)	0.572	765 (57.5)	750 (55.1)	0.207
Creatinine, mean ± SD	2.3 ± 1.9	2.4 ± 1.7	0.744	0.8 ± 0.2	0.8 ± 0.2	< 0.0001*
**Baseline eGFR, N (%)**			0.092			–
eGFR 30–59 mL/min/1.73 m^2^	590 (61.7)	623 (57.0)		–	-	
eGFR 15–29 mL/min/1.73 m^2^	209 (21.8)	275 (25.2)		–	–	
eGFR < 15 mL/min/1.73 m^2^	158 (16.5)	195 (17.8)		–	–	
**Comorbidities before the index date, N (%)**						
Hypertension	449 (46.9)	515 (47.1)	0.928	400 (30.1)	359 (26.4)	0.033*
Heart disease	466 (48.7)	509 (46.6)	0.337	345 (25.9)	314 (23.1)	0.084
Diabetes mellitus	405 (42.3)	459 (42.0)	0.882	337 (25.3)	356 (26.2)	0.627
Anemia	127 (13.3)	144 (13.2)	0.949	71 (5.3)	108 (7.9)	0.007*
Cerebrovascular disease	233 (24.3)	273 (25.0)	0.741	323 (24.3)	261 (19.2)	0.001*
Malignancy	142 (14.8)	185 (16.9)	0.198	415 (31.2)	558 (41.0)	< 0.0001*
COPD	104 (10.9)	133 (12.2)	0.358	163 (12.3)	131 (9.6)	0.029*
Digestive tract disease	504 (52.7)	575 (52.6)	0.979	728 (54.7)	716 (52.6)	0.268
**Blood electrolytes**						
Sodium, mEq/L, mean ± SD	137.9 ± 8.3	138.2 ± 8.3	0.390	137.3 ± 7.7	137.1 ± 8.0	0.563
Potassium, mEq/L, mean ± SD	4.3 ± 1.0	4.3 ± 0.9	0.417	3.9 ± 0.8	4.0 ± 0.8	0.859

*P < 0.05.

*SD* standard deviation, *eGFR* estimated glomerular filtration rate, *AKI* acute kidney injury, *AKD* acute kidney disease, *COPD* chronic obstructive pulmonary disease.

### Severe AKD was associated with adverse kidney outcomes among the lower baseline eGFR and higher baseline eGFR populations

**Figure 3:**
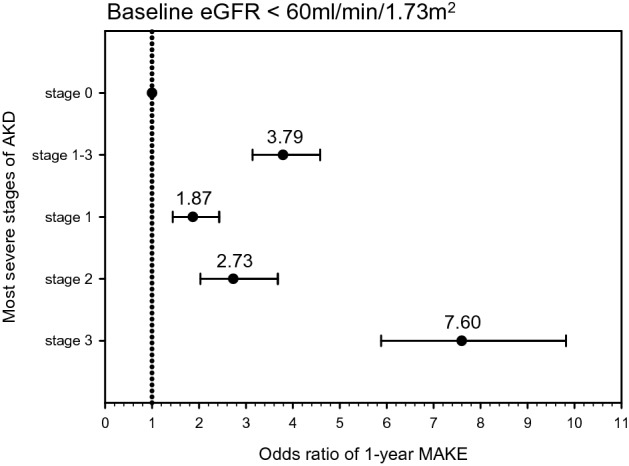
Adjusted odds of major adverse kidney event at 1-year follow-up in lower baseline eGFR group. Odds of major adverse kidney events stratified by most severe stage of AKD between 7–90 days after AKI, according to 16th Acute Disease Quality Initiative (ADQI) recommendations, in lower baseline eGFR group. Adjusted odds ratios (dots) and 95% CIs (lines) were calculated using logistic regression, where the reference category was patients who were AKD stage 0. Logistic regression was adjusted for age, sex, hypertension, heart disease, diabetes, anemia, cerebrovascular disease, cancer, COPD and digestive tract disease. Clinical significance was observed while comparing AKD stages 1–3 versus stage 0. *AKD* acute kidney disease, *AKI* acute kidney injury, *eGFR* estimated glomerular filtration rate, *COPD* chronic obstructive pulmonary disease.

**Figure 4:**
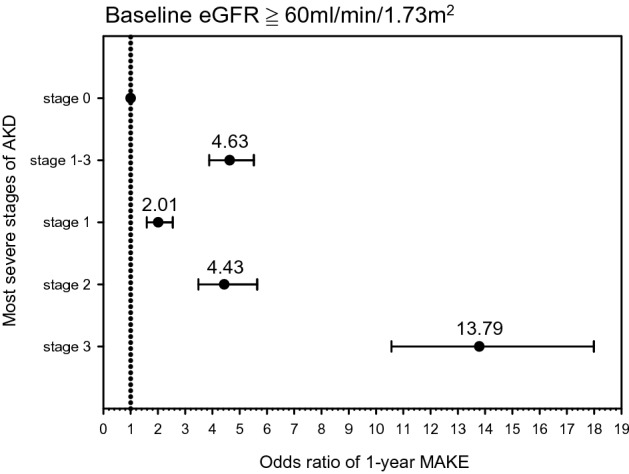
Adjusted odds of major adverse kidney event at 1-year follow-up in higher baseline eGFR group. Odds of major adverse kidney events stratified by most severe stage of AKD between 7–90 days after AKI, according to 16th Acute Disease Quality Initiative (ADQI) recommendations, in higher baseline eGFR group. Adjusted odds ratios (dots) and 95% CIs (lines) were calculated using logistic regression, where the reference category was patients who were AKD stage 0. Logistic regression was adjusted for age, sex, hypertension, heart disease, diabetes, anemia, cerebrovascular disease, cancer, COPD and digestive tract disease. Clinical significance was observed while comparing AKD stages 1–3 versus stage 0. *AKD* acute kidney disease, *AKI* acute kidney injury, *eGFR* estimated glomerular filtration rate, *COPD* chronic obstructive pulmonary disease.

Among the patients with lower baseline eGFR, a year after AKI, those who had AKD stage 1–3 were noted to have higher risk of MAKEs (a composite outcome containing rapid eGFR decline, initiation of dialysis, and in-hospital mortality; see the “Follow-up and study outcomes” subsection of the “Methods” section) after adjustment for age, sex, and comorbidities (odds ratio [OR] 3.79; CI 3.4–4.58) compared with those who had AKD stage 0 (Fig. 3). Similarly, among the higher baseline eGFR patients, those who had AKD stage 1–3 were more likely to develop MAKEs a year after AKI (OR 4.63, CI 3.88–5.52) (Fig. 4). Regarding the individual components of MAKE, AKD stage 1–3 was significantly associated with rapid eGFR decline at 1 year among the lower baseline eGFR population (OR 2.53, CI 1.91–3.35) and the higher baseline eGFR population (OR 2.55, CI 1.92–3.37) (Table S3 and Table S4). As to the initiation of kidney replacement therapy (KRT), among the lower baseline eGFR population, AKD stage 1–3 was positively associated with KRT commencement (OR 3.29, CI 2.63–4.11). Similarly, in the higher baseline eGFR group, AKD stage 1–3 was significantly associated with KRT initiation (OR 10.47, CI 5.97–18.36). AKD stage 1–3 was associated with a higher 1-year in-hospital mortality rate in the lower baseline eGFR group (OR 2.64, CI 2.14–3.27) and the higher baseline eGFR group (OR 4.04, CI 3.33–4.89).

### More advanced AKD stages correspond to worse kidney outcomes

The association between different AKD stages and adverse kidney outcomes was analyzed to determine the clinical significance of AKD stage. At the 1-year follow-up, compared with patients in the AKD stage 0 group, those with all other AKD stages were associated with a higher risk of MAKEs (AKD stage 1: OR 1.85, CI 1.56–2.19; AKD stage 2: OR 3.45, CI 2.85–4.12; AKD stage 3: OR 10.41, CI 8.68–12.49) (Table 2). Notably, all AKD stages were associated with MAKEs at 1 year for both the lower baseline eGFR group and the higher baseline eGFR group (Figs. 3 and 4). In addition, rapid eGFR decline was associated with different AKD stages, both in the lower and higher baseline eGFR groups. AKD stages 2 and 3 were associated with KRT initiation, whereas AKD stage 1 was not (AKD stage 1: OR 1.01, CI 0.74–1.39; AKD stage 2: OR 1.88, CI 1.39–2.53; AKD stage 3: OR 8.72, CI 7.07–10.76) (Table 2). Furthermore, in both the lower baseline eGFR group and the higher baseline eGFR group, only AKD stages 2 and 3 were associated with KRT initiation (Table S3 and Table S4). Compared with the AKD stage 0 group, all other AKD stages were associated with a higher risk of in-hospital mortality in both the lower baseline eGFR group and the higher baseline eGFR group (Table S3 and Table S4).

**Table 2:** Association between AKD stage and major adverse kidney events among all patients.

AKD stages	MAKE			eGFR decline			KRT			In-hospital mortality		
	Adjusted regression			Adjusted regression			Adjusted regression			Adjusted regression		
	No of Events	Coefficient	95% CI	No of Events	Coefficient	95% CI	No of Events	Coefficient	95% CI	No of Events	Coefficient	95% CI
AKD 0	614	1.00	Reference	156	1.00	Reference	174	1.00	Reference	376	1.00	Reference
AKD 1	330	1.85*	1.56–2.19	108	2.14*	1.65–2.79	57	1.01*	0.74–1.39	219	1.79*	1.47–2.18
AKD 2	358	3.43*	2.85–4.12	100	2.64*	2.01–3.47	72	1.88*	1.39–2.53	258	3.23*	2.65–3.94
AKD 3	783	10.41*	8.68–12.49	180	2.90*	2.29–3.66	400	8.72*	7.07–10.76	488	5.59*	4.69–6.67

Adjustment: age, sex, hypertension, heart disease, diabetes, anemia, cerebrovascular disease, cancer, COPD and digestive tract disease.

*AKD* acute kidney disease, *MAKE* major adverse kidney event, *eGFR* estimated glomerular filtration rate, *KRT* kidney replacement therapy.

*P < 0.05.

### Severe AKD is associated with an increased risk of prolonged dialysis

Persistent kidney function impairment can cause irreversible kidney damage and long-term dialysis dependency among patients with lower baseline eGFR. Notably, in patients with lower baseline eGFR, hypertension and diabetes were risk factors for chronic dialysis at the 1-year follow-up. However, old age and cerebrovascular disease were associated with a lower risk of prolonged dialysis. After adjustment, AKD stage 1–3 was noted to be associated with a higher risk of prolonged dialysis (OR 2.16, CI 1.45–3.23), in comparison with AKD stage 0 group. However, not all AKD stages were noted to be risk factors, and only AKD stage 3 was significantly associated with prolonged dialysis (Table S3).

## Discussion

In our longitudinal follow-up study involving 4741 AKI patients, all the patients were considered as having AKD by the current KDIGO consensus^15^. In both the lower baseline eGFR group and the higher baseline eGFR group, more than 50% of the patients had stage 1–3 AKD. The dauntingly high incidence of severe AKD matches with previous studies. In a multicenter retrospective cohort study conducted by Xiao et al., 53.17% (1359/2556) of the AKI patients developed AKD^16^. In a single center 10-year follow-up study by Nagata et al., AKD patient accounted for 66.8% of all AKI patients^14^.

Although a definition of AKD was proposed in the KDIGO AKI guidelines in 2012, limited data have been published regarding the clinical significance of AKD^7,10,21^. The KDIGO definition of AKD comprises the following criteria with the same timeline of less than 3 months: (1) AKI (congruent with AKI), or (2) GFR < 60 mL/min/1.73 m^2^ (approximating CKD), or (3) decrease in GFR by > 35%, or increase in SCr by > 50% (rapid progression of renal dysfunction between AKI and CKD)^12,15^. James et al. separated patients with post-AKI AKD and patients with AKD without AKI. After a 10-year follow-up of a 1-year regional cohort, they successfully demonstrated the clinical significance of AKD without AKI. Notably, compared with patients without kidney disease, those with AKD without AKI had higher risks of mortality, ESKD, and development or progression of CKD^10^. However, this study involving a retrospective cohort did not address the severity of AKI and post-AKI AKD, and thus, it could not provide information regarding the risk stratification among AKI patients. In a recent comparative analysis by See et al., among all the 36,118 hospitalized adults with a baseline eGFR ≥ 60 mL/min/1.73 m^2^, AKI, AKD with AKI and AKD without AKI were all associated with worse kidney outcome and higher mortality risk^11^. Interestingly, in keeping with our findings, multivariable Cox regression analysis of the risk of MAKEs found that the hazard ratios for MAKE of AKI alone group and AKD with AKI (post-AKI AKD) group were widely separated, which may represent significantly different risk between two groups [AKI alone, HR 1.52 (95% CI 1.35–1.72) VS AKD with AKI, HR 2.51 (95% CI 2.16–2.91)]. In the present study, we furtherly proved that post-AKI AKD was associated with worse clinical outcome, not only in hospitalized adults with a baseline eGFR ≥ 60 mL/min/1.73 m^2^, but also in those with baseline eGFR < 60 mL/min/1.73 m^2^ (lower baseline eGFR group) across all clinical settings (all EMR data in our analysis comprised data from hospitalized patients and outpatient clinic patients).

Different stages of post-AKI AKD have diverse clinical outcomes. Notably, international experts have achieved a consensus regarding AKD staging^2^, recommending a clinical classification based on SCr data. The criteria of post-AKI AKD stages are congruent with the KDIGO guidelines on AKI stages. Chen et al. studied a cohort of patients on extracorporeal membrane oxygenation and observed that AKD stages were independent risk factors for mortality^22^. In the present study, we further delineated the prognostic effects of different AKD stages related to various MAKEs. Regarding MAKEs at 1 year, a more severe AKD stage was noted to be associated with worse kidney outcomes. Moreover, the risk of 1-year MAKEs seemed to increase in direct proportion with AKD stage. AKD was also associated with an increased risk of in-hospital mortality, both in the lower baseline eGFR and the higher baseline eGFR groups.

Compared with the AKD stage 0 group (SCr returning to lower than 1.5-fold of baseline value between 7 and 90 days after AKI), the group with all other AKD stages (AKD stages 1, 2, and 3) had a higher probability of eGFR decline of more than 35% beyond 90 days after AKI. Moreover, non-recovery of kidney dysfunction within 7–90 days after AKI reasonably predicted persistent kidney damage beyond 90 days after AKI and the development or deterioration of CKD. Heung et al. conducted a retrospective study using a large administrative database of the veterans’ health system, in which also found that the persistently elevated SCr after AKI was associated with the development of CKD within 1 year, regardless of AKI severity^23^.

Regarding KRT initiation, only patients with AKD stages 2 and 3 had a higher risk of requiring KRT. It was also noted that only AKD stage 3 was associated with prolonged dialysis. This finding precisely indicates that the AKD stages should be congruent with AKI stages, with one of the criteria of stage 3 AKI being “dialysis-requiring AKI (AKI-D)”. The present study confirms that stage 3 AKD is likely to progress to “dialysis-requiring AKD (AKD-D)”. Therefore, patients with AKD with either “SCr increased to more than 3 times of the baseline SCr” or “unresolved dependency on dialysis” should be categorized as having stage 3 AKD (AKD-3).

There are several limitations in our study. First, in our algorithm for the definition of acute kidney injury (AKI) (Fig. S1), we adopted NHS England AKI algorithm and used two strategies to define baseline SCr. We first searched for the lowest SCr within 0–7 days before the index SCr as baseline. If such an baseline SCr were not available, we then searched median value of all available SCr data within 8–365 days of the index as reference SCr. Different definitions of baseline SCr may result into higher or lower rate of AKI diagnosis^24^. However, previous studies have validated the robustness of such algorithm^25,26^. The AKI events defined by the NHS England AKI algorithm could also be used as gold-standard in the machine learning prediction study^27^. Second, we retrieved the maximum SCr value between 7 and 90th day after AKI for AKD staging. Within this post-AKI time period, AKI recovery and multiple AKI episodes might occur. Also, dialysis might affect the SCr value during post-AKI period. However, whether recurrent AKI or a very high pre-dialysis SCr may be all considered as “non-recovery” during the post-AKI period. Severe AKD (AKD stage 1–3) by SCr still represents the persistence or the aggravation of kidney dysfunction following AKI. Third, many patients with AKI had no SCr data between 7 and 90 days after AKI for defining and staging AKD. The proportion of missing data within this period was markedly high, at approximately 43% (3781 patients out of 8718 patients). The lack of follow-up SCr data might be attributed to early mortality, early recovery from AKI, and most likely unawareness of the occurrence and significance of AKI. A recent analysis by Wu et al. in Taiwan revealed that only 37% of patients with dialysis-requiring AKI (AKI-D) weaned from dialysis received the nephrologist follow-up during the AKD period^28^. AKI electronic alert (AKI eAlert) systems^29–31^, might increase the awareness of AKI and facilitate follow-up SCr data generation and help reduce the hospital stay duration when it is coupled with care bundle^32^.

Proteinuria has been noted to be an independent risk factor for predicting the development and severity of AKI among surgical and hospitalized patients^33–37^. Recently, Hsu et al. demonstrated the significance of post-AKI proteinuria in a multicenter, prospective cohort study of patients with AKI, wherein a higher post-AKI urinary albumin-to-creatinine ratio (ACR) was noted to be associated with rapid kidney disease progression^7^. Notably, after adjustment for the ACR and traditional clinical kidney disease risk factors, AKI stages were not observed to be independently associated with adverse clinical outcomes. In our longitudinal follow-up study, baseline proteinuria data were available for only approximately 18% of patients with AKI. Therefore, proteinuria level cannot be used either as a baseline characteristic to define CKD status or clinical predictor for renal disease progression. Regarding the severity of kidney disease, namely the stages of post-AKI AKD, the present study observed a significant association between different AKD stages and various adverse clinical outcomes. Furthermore, more severe AKD stages were noted to be associated with higher risks of in-hospital mortality, renal disease progression, and need for dialysis.

Second, local regulations do not permit the linkage of our single-hospital database with the National Health Insurance Research Database (NHIRD). Therefore, certain adverse clinical events might have occurred at another hospital without being documented in our health information system.

In conclusion, after AKI, we found that AKD stage 1–3 were common among AKI patients. Post-AKI AKD is associated with an increased risk of eGFR decline, need for KRT, and in-hospital mortality among patients with lower and higher baseline eGFR. Moreover, AKD is associated with a higher risk of requiring prolonged dialysis among patients with baseline eGFR < 60 mL/min/1.73 m^2^. Therefore, timely intervention with intensified care program shall be established and embed into the health care system to stop the AKI-AKD-CKD continuum.

## Supplementary Information

Supplementary Information.

## Data Availability

The data underlying this article will be shared on reasonable request to the corresponding author.
