# Supplementary material for: Severe acute kidney disease is associated with worse kidney outcome among acute kidney injury patients
Source: Sci Rep. 2022 Apr 20;12:6492. doi: 10.1038/s41598-022-09599-7 (PMC9021248; doi:10.1038/s41598-022-09599-7)
Supplement: Supplementary file 1 — Supplementary Information. [file 41598_2022_9599_MOESM1_ESM.pdf]

Severe Acute Kidney Disease Is Associated With Worse Kidney Outcome Among Acute Kidney Injury Patients

Yu-Wei Chen, MD[1,2,3], Mei-Yi Wu, MD[1,3,4,5], Cheng-Hsien Mao[6], Yu-Ting Yeh, PhD[6], Tzu-Ting Chen, PhD[4,7], Chia-Te Liao, MD, PhD[1,3,5], Cai-Mei Zheng, MD, PhD[1,3,5], Yung-Ho Hsu, MD, MS[1,2,3,5], Yih-Giun Cherng, MD, MBA[8,9*], and Mai-Szu Wu, MD [1,2,3,5*]

[1]Division of Nephrology, Department of Internal Medicine, Shuang Ho Hospital, Taipei Medical University, New Taipei City, Taiwan

[2]Graduate Institute of Clinical Medicine, College of Medicine, Taipei Medical

University, Taipei, Taiwan

[3]TMU Research Center of Urology and Kidney, Taipei Medical University, Taipei, Taiwan

[4]Institute of Epidemiology and Preventive Medicine, College of Public Health, National Taiwan University, Taipei, Taiwan

[5]Division of Nephrology, Department of Internal Medicine, School of Medicine, College of Medicine, Taipei Medical University, Taipei, Taiwan

[6]Information Technology Office, Shuang Ho Hospital, Taipei Medical University, Taipei, Taiwan

[7]Center for Neuropsychiatric Research, National Health Research Institutes, Miaoli County,

Taiwan

[8]Department of Anesthesiology, Shuang Ho Hospital, Taipei Medical University, New Taipei City, Taiwan

[9]Department of Anesthesiology, School of Medicine, College of Medicine, Taipei Medical University, Taipei, Taiwan

* Corresponding authors, equal contribution

Correspondence:

Mai-Szu Wu, MD

Division of Nephrology, Department of Internal Medicine, Shuang Ho Hospital, Taipei Medical University, Taipei, Taiwan

No. 291, Zhongzheng Road, Zhonghe District, New Taipei City, Taiwan 235

Tel: +886-2-22490088 #2717

Email: maiszuwu@gmail.com

List of Supplementary Material

Figure S1. Algorithm for the definition of acute kidney injury

Table S1. Characteristics of lower baseline eGFR patients with different AKD stages

Table S2. Characteristics of higher baseline eGFR patients with different AKD stages

Table S3. Association between AKD stage and major adverse kidney events among lower baseline eGFR patients

Table S4. Association between AKD stage and major adverse kidney events among higher baseline eGFR patients

Supplement Figures Legends

Figure S1. Algorithm for the definition of acute kidney injury (AKI), modified from the 2014 NHS England “algorithm for detecting acute kidney injury (AKI) based on serum creatinine changes with time. SCr, serum creatinine; RV, reference value. Only patients with AKI stage 1-3 by this algorithm were defined as AKI patients and included for further analysis. Conversion factors for serum creatinine in mg/dL to μmol/L, ×88.4.

Figure S1. Algorithm for the definition of acute kidney injury

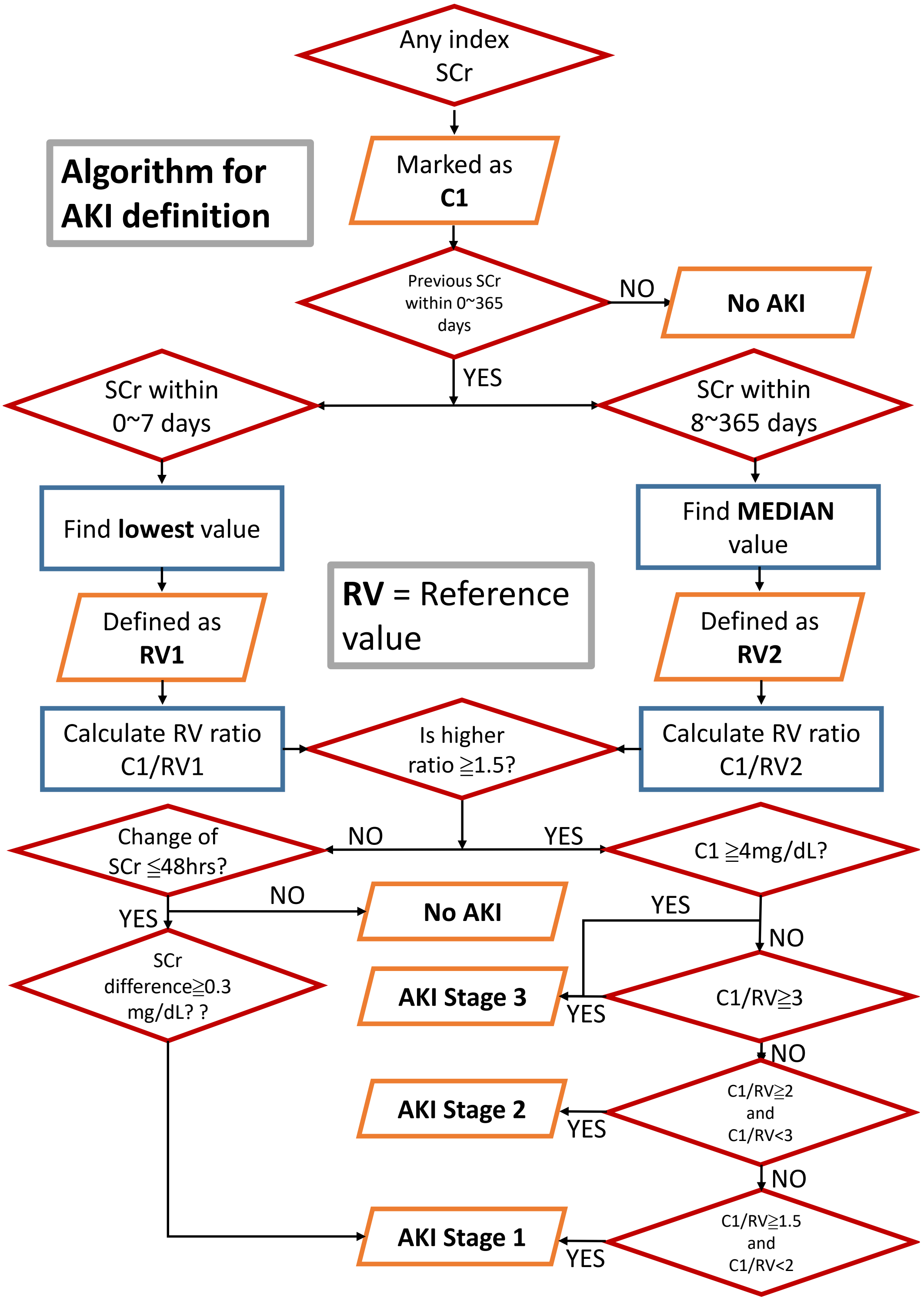

Table S1. Characteristics of lower baseline eGFR patients with different AKD stages

	Lower baseline eGFR				
	AKI				
	**AKD stage 0**	**AKD stage 1**	**AKD stage 2**	**AKD stage 3**	**P value**
N	957	313	236	544	
Age, y, mean ± SD	74.9 ± 13.1	76.2 ± 12.6	76.8 ± 13.1	72.1 ± 14.9	<0.001*
Age group, y, N (%)					0.003*
20-29	6 (0.6)	0 (0.0)	0 (0.0)	5 (0.9)	
30-39	10 (1.0)	3 (0.9)	1 (0.4)	15 (2.8)	
40-49	26 (2.7)	9 (2.9)	10 (4.2)	23 (4.2)	
50-59	71 (7.4)	21 (6.7)	16 (6.8)	55 (10.1)	
60-69	179 (18.7)	50 (16.0)	40 (16.9)	106 (19.5)	
70-79	267 (27.9)	89 (28.4)	46 (19.5)	138 (25.4)	
≥80	398 (41.6)	141 (45.0)	123 (52.1)	202 (37.1)	
Male sex, N (%)	502 (52.5)	174 (55.6)	114 (48.3)	299 (55.0)	0.275
Creatinine, mean ± SD	2.3 ± 1.9	2.1 ± 1.5	1.9 ± 1.4	2.8 ± 1.8	<0.001*

Baseline eGFR, N (%)					<0.001*
eGFR 30~59ml/min/1.73m^2	590 (61.7)	212 (67.7)	178 (75.4)	233 (42.8)	
eGFR 15~29 ml/min/1.73m^2	209 (21.8)	62 (19.8)	40 (16.9)	173 (31.8)	
eGFR < 15ml/min/1.73m^2	158 (16.5)	39 (12.5)	18 (7.6)	138 (25.4)	
**Comorbidities before the index date, N (%)**					
Hypertension	449 (46.9)	145 (46.3)	97 (41.1)	273 (50.2)	0.135
Heart disease	466 (48.7)	145 (46.3)	102 (43.2)	262 (48.2)	0.467
Diabetes mellitus	405 (42.3)	135 (43.1)	79 (33.5)	245 (45.0)	0.026*
Anemia	127 (13.3)	28 (8.9)	23 (9.7)	93 (17.1)	0.002*
Cerebrovascular disease	233 (24.3)	84 (26.8)	69 (29.2)	120 (22.1)	0.139
Malignancy	142 (14.8)	50 (16.0)	39 (16.5)	96 (17.6)	0.550
COPD	104 (10.9)	39 (12.5)	33 (14.0)	61 (11.2)	0.549
Digestive tract disease	504 (52.7)	166 (53.0)	125 (53.0)	284 (52.2)	0.995
**Blood electrolytes**					
Sodium, mEq/L, mean ± SD	137.9 ± 8.3	138.4 ± 8.1	138.4 ± 8.0	138.0 ± 8.6	0.738

Potassium, mEq/L, mean ± SD	4.3 ± 1.0	4.3 ± 0.9	4.2 ± 0.9	4.4 ± 0.9	0.048*

*$P<0.05$

SD: standard deviation; CKD: Chronic kidney disease; AKI: Acute kidney injury; AKD: Acute kidney disease; COPD: Chronic obstructive pulmonary disease

Table S2. Characteristics of higher baseline eGFR patients with different AKD stages

	Higher baseline eGFR				
	AKI				
	**AKD stage 0**	**AKD stage 1**	**AKD stage 2**	**AKD stage 3**	**P value**
N	1330	499	414	448	
Age, y, mean ± SD	65.7 ± 15.3	64.6 ± 15.2	65.6 ± 14.5	64.7 ± 16.2	0.394
Age group, y, N (%)					0.218
20-29	20 (1.5)	6 (1.2)	2 (0.5)	13 (2.9)	
30-39	50 (3.8)	23 (4.6)	10 (2.4)	16 (3.6)	
40-49	136 (10.2)	50 (10.0)	43 (10.4)	50 (11.2)	
50-59	241 (18.1)	106 (21.2)	93 (22.5)	88 (19.6)	
60-69	314 (23.6)	131 (26.3)	97 (23.4)	96 (21.4)	
70-79	288 (21.7)	88 (17.6)	86 (20.8)	90 (20.1)	
≥80	281 (21.1)	95 (19.0)	83 (20.0)	95 (21.2)	
Male sex, N (%)	765 (57.5)	267 (53.5)	228 (55.1)	255 (56.9)	0.439
Creatinine, mean ± SD	0.8 ± 0.2	0.7 ± 0.2	0.8 ± 0.2	0.8 ± 0.2	<0.001*

**Comorbidities before the index date, N (%)**					
Hypertension	400 (30.1)	142 (28.5)	113 (27.3)	104 (23.2)	0.046*
Heart disease	345 (25.9)	127 (25.5)	82 (19.8)	105 (23.4)	0.074
Diabetes mellitus	337 (25.3)	123 (24.6)	116 (28.0)	117 (26.1)	0.664
Anemia	71 (5.3)	35 (7.0)	27 (6.5)	46 (10.3)	0.004*
Cerebrovascular disease	323 (24.3)	99 (19.8)	88 (21.3)	74 (16.5)	0.004*
Malignancy	415 (31.2)	218 (43.7)	180 (43.5)	160 (35.7)	<0.001*
COPD	163 (12.3)	53 (10.6)	43 (10.4)	35 (7.8)	0.070
Digestive tract disease	728 (54.7)	246 (49.3)	230 (55.6)	240 (53.6)	0.169
**Blood electrolytes**					
Sodium, mEq/L, mean ± SD	137.3 ± 7.7	137.3 ± 6.7	137.1 ± 8.4	136.9 ± 8.9	0.856
Potassium, mEq/L, mean ± SD	3.9 ± 0.8	3.9 ± 0.8	3.9 ± 0.8	4.0 ± 0.9	0.190

*$P<0.05$

SD: standard deviation; CKD: Chronic kidney disease; AKI: Acute kidney injury; AKD: Acute kidney disease; COPD: Chronic obstructive pulmonary disease

Table S3. Association between AKD stage and major adverse kidney events among lower baseline eGFR patients

	eGFR decline Adjusted regression			KRT Adjusted regression			In-hospital mortality Adjusted regression			Prolonged dialysis Adjusted regression		
**AKD stages**	**No of Events**	**Coefficient**	**95% CI**	**No of Events**	**Coefficient**	**95% CI**	**No of Events**	**Coefficient**	**95% CI**	**No of Events**	**Coefficient**	**95% CI**
AKD 0	79	1.00	Reference	160	1.00	Reference	177	1.00	Reference	38	1.00	Reference
AKD 1-3 (pooled)	198	2.53*	1.91-3.35	401	3.29*	2.63-4.11	398	2.64*	2.14-3.27	86	2.16*	1.45-3.23
AKD 1	42	1.82*	1.21-2.73	55	1.21	0.85-1.71	97	1.96*	1.45-2.64	8	0.69	0.35-1.51
AKD 2	32	1.99*	1.27-3.11	53	1.87*	1.29-2.70	90	2.68*	1.95-3.70	6	0.82	0.34-2.00
AKD 3	124	3.18*	2.33-4.35	293	6.27*	4.85-8.10	211	3.12*	2.43-4.00	72	3.48*	2.29-5.31

Adjustment: age, sex, hypertension, heart disease, diabetes, anemia, cerebrovascular disease, cancer, COPD and digestive tract disease.

Abbreviations: AKD, acute kidney disease; eGFR, estimated glomerular filtration rate; KRT, kidney replacement therapy; COPD, chronic obstructive pulmonary disease

*$P < 0.05$

Table S4. Association between AKD stage and major adverse kidney events among higher baseline eGFR patients

	eGFR decline Adjusted regression			KRT Adjusted regression			In-hospital mortality Adjusted regression			Prolonged dialysis Adjusted regression		
**AKD stages**	**No of Events**	**Coefficient**	**95% CI**	**No of Events**	**Coefficient**	**95% CI**	**No of Events**	**Coefficient**	**95% CI**	**No of Events**	**Coefficient**	**95% CI**
AKD 0	77	1.00	Reference	14	1.00	Reference	199	1.00	Reference	0	NA	
AKD 1-3 (pooled)	190	2.55*	1.92-3.37	128	10.47*	5.97-18.36	567	4.04*	3.33-4.89	6	NA	
AKD 1	66	2.41*	1.70-3.43	2	0.40	0.09-1.78	122	1.72*	1.32-2.23	0	NA	
AKD 2	68	3.14*	2.21-4.47	19	5.05*	2.49-10.25	168	3.74*	2.89-4.83	0	NA	
AKD 3	56	2.18*	1.51-3.16	107	31.43*	17.60-56.11	277	10.48*	8.09-13.57	6	NA	

Adjustment: age, sex, hypertension, heart disease, diabetes, anemia, cerebrovascular disease, cancer, COPD and digestive tract disease.

Abbreviations: AKD, acute kidney disease; eGFR, estimated glomerular filtration rate; KRT, kidney replacement therapy; COPD, chronic obstructive pulmonary disease; NA: not available

*$P < 0.05$
